# Neuron matters: neuromodulation with electromagnetic stimulation must consider neurons as dynamic identities

**DOI:** 10.1186/s12984-022-01094-4

**Published:** 2022-11-03

**Authors:** Hui Ye, Jenna Hendee, Joyce Ruan, Alena Zhirova, Jayden Ye, Maria Dima

**Affiliations:** Department of Biology, Quinlan Life Sciences Education and Research Center, Loyola University Chicago, 1032 W. Sheridan Rd., Chicago, IL 60660 USA

**Keywords:** Neuromodulation, Electrical stimulation, Neuron, Dynamics, Ion channels, Deep brain stimulation (DBS), Transcranial magnetic stimulation (TMS)

## Abstract

Neuromodulation with electromagnetic stimulation is widely used for the control of abnormal neural activity, and has been proven to be a valuable alternative to pharmacological tools for the treatment of many neurological diseases. Tremendous efforts have been focused on the design of the stimulation apparatus (i.e., electrodes and magnetic coils) that delivers the electric current to the neural tissue, and the optimization of the stimulation parameters. Less attention has been given to the complicated, dynamic properties of the neurons, and their context-dependent impact on the stimulation effects. This review focuses on the neuronal factors that influence the outcomes of electromagnetic stimulation in neuromodulation. Evidence from multiple levels (tissue, cellular, and single ion channel) are reviewed. Properties of the neural elements and their dynamic changes play a significant role in the outcome of electromagnetic stimulation. This angle of understanding yields a comprehensive perspective of neural activity during electrical neuromodulation, and provides insights in the design and development of novel stimulation technology.

## Introduction

As electrically excitable cells, neurons transfer information among each other using synapses or electric connections. Fluctuations of the transmembrane potential and dynamic changes in the voltage-dependent ion channels produce action potentials and induce synaptic connections with other neurons. These unique features of the neurons, therefore, allow the exogenous electrical signals to control or modify their excitability, behavior, and functions. Numerous electrical stimulation devices have been invented to deliver electric currents that target the brain and spinal cord for neuromodulation. Among these many clinical successes, deep brain stimulation (DBS) uses implanted electrode leads. DBS is an established symptomatic surgical therapy for Parkinson’s disease, essential tremor, and several other movement and neuropsychiatric disorders [1]. Transcranial magnetic stimulation (TMS), an electromagnetic technology, induces electric currents to control neural activities inside the brain [2]. Transcranial direct-current stimulation (tDCS) uses weak, direct currents to shift the resting potential of cortical neurons in a non-invasive manner. Spinal cord stimulation (SCS) uses minimally invasive stimulation for the treatment of chronic neuropathic pain [3]. From the start of these clinical practices with electrical neuromodulation, researchers began to ask the questions, “What determines the outcome of electrical stimulation? How can we improve it?”.

From the early years of its practice, researchers have realized that optimal control of neural activity with the electric field seemed largely dependent on the properties of the electric currents that were applied to the target neuronal tissue. Many key parameters that define the electric current have been exclusively studied, such as intensity, duration, frequency, and orientation [4–6]. For example, neurons in the motor cortex displayed different sensitivities to transcranial magnetic fields with differing coil orientations, shapes of the induced current pulse, and intensities [7–12]. Development of various technologies has allowed researchers to link various stimulation parameters to fundamental cellular physiology, such as the excitation of individual neurons [13], changes in ion channel dynamics [14], and alternation of synaptic transmission [15, 16], etc. Cellular analysis reveals that the orientation of the magnetic field determines the geometric pattern of membrane polarization on the neuron [13, 17]. To achieve optimal outcomes, clinical research witnesses the continuous optimization of the stimulating parameters [18–20]. In TMS, with careful coil design and placement around the head, the magnetic field can eventually achieve improved clinical outcomes [10, 11].

Even though substantial variance has been observed in patients and normal subjects, at the individual neuron level, the impacts of the targeted tissue and neurons on the outcome of the electrical stimulation has drawn far less attention. For example, repetitive transcranial magnetic stimulation (rTMS) over the occipital cortex led to a significantly increased visual cortex excitability in subjects affected by migraine with aura, but a decreased visual cortex excitability in normal subjects [21]. At the cellular level, the same electrical stimulation protocol can cause significant difference in neural activity. Identical stimulation parameters can result in neuronal activation, suppression, or both, depending on the brain region [22]. Considering the complicated interactions between the applied electric field and the biological tissue (reviewed in [23]), it is essential to understand the outcome of electrical stimulation and its dependence on both the properties of the stimuli and its neural targets.

**Fig. 1:**
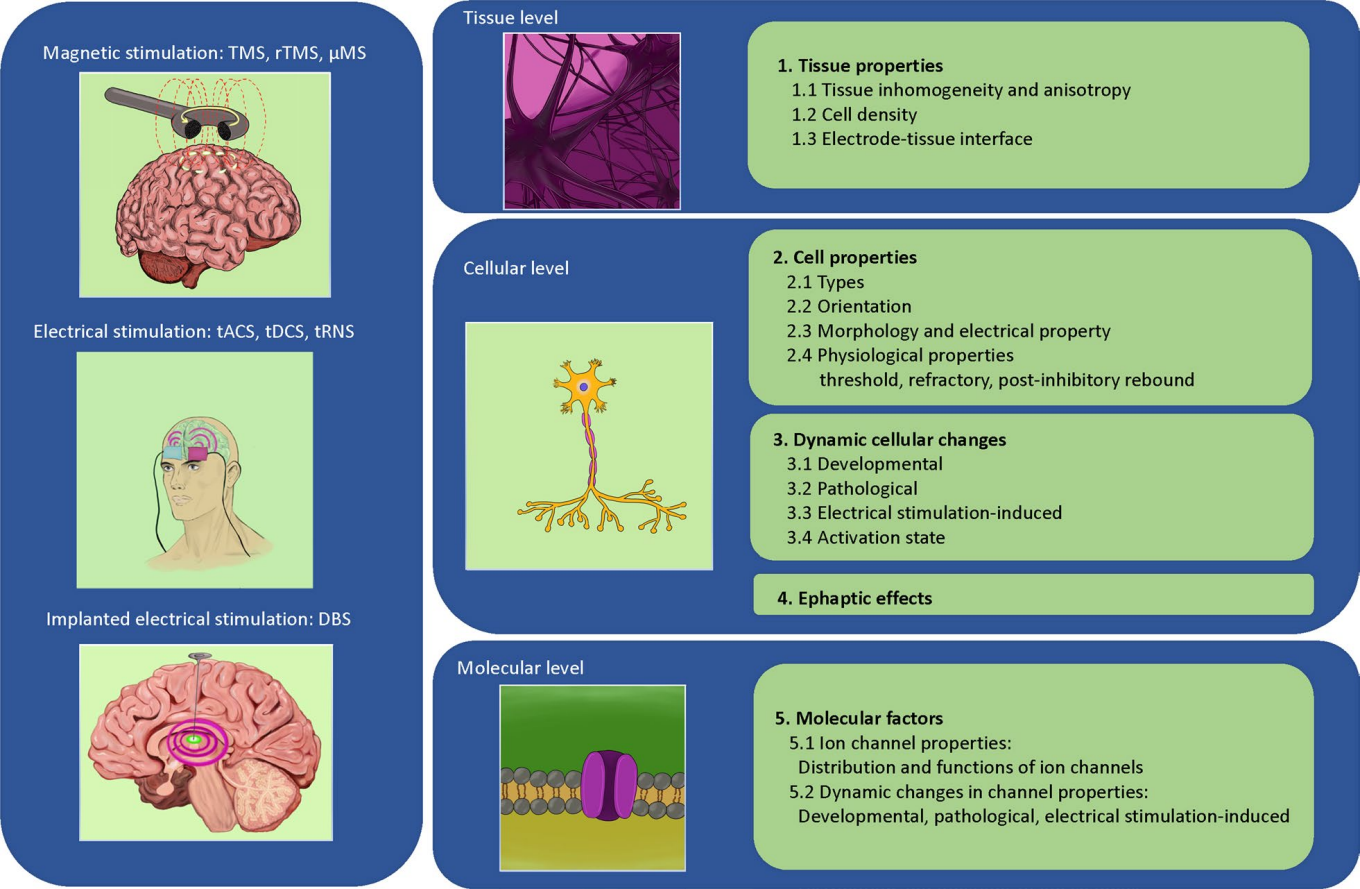
Conceptual figure that summarizes multiple different electromagnetic stimulation modalities across different biological levels. The text of the paper is organized to provide evidence for how the different factors at each of these levels could impact the outcome of the stimulation. Abbreviations are provided separately in the “abbreviations list” of the text

This paper will review the impact of biological complexity on the outcomes of neuromodulation with electromagnetic stimulation. We organize our evidence in a multidimensional format—at the tissue level, cell level, and molecular/ion channel level, respectively (Fig. 1). It is our hope that reviewing these biological complexities during electrical stimulation will provoke awareness in the scientific community during the investigation of the underlying mechanisms of stimulation, and will facilitate the design and development of novel protocols and technologies for neural control with electrical stimulation.

## Review

### Dynamic changes in tissue properties impact neural stimulation with electromagnetic fields

Biophysical complexity of the neural tissue that surrounds the stimulating electrode alters the electric current distribution and the stimulation outcome. This includes, but is not limited to, inhomogeneity and anisotropy of the tissue, compactness of the cell, and impedance change due to electrode-tissue interaction.

#### Impact of tissue inhomogeneity and anisotropy on electrical stimulation

Under electrical stimulation, distribution of the electric current inside the neural tissue is largely dependent on the electrical properties of the tissue, including homogeneity and isotropy. A homogeneous material has the same properties at every point. An isotropic material has the same physical properties in all directions. Most neural tissues are inhomogeneous, compositing different types of cells, tissues, fat, irregular vascularization, pH variation, and heterogeneous oxygen concentration, etc. Inhomogeneous resistance is present in the extracellular space in many brain regions, such as the hippocampus and the cerebellum [24]. For example, the tissue conductivity can vary in different layers of the hippocampus [25], with the resistance in the stratum pyramidale two times higher than other hippocampal layers. Additionally, tissue swelling has been observed after intense neural activity, which could lead to an increase in extracellular resistance [26]. Similarly, directional variance in electrophysical properties (anisotropy) is frequently observed in biological tissue. This material property allows tissues to change or assume different properties in different directions. For example, the specific direction of a fiber track generates anisotropy in the brain [27].

Under electrical stimulation, the electric field could be significantly dispersed by non-homogenous tissue conductivity and anisotropy. A recent numerical study [28] suggested that a decrease in tissue conductivity resulted in a smaller volume of neuronal tissue being activated by an electrode. Anisotropic properties of the head play significant roles in the distribution of the electric fields and the effects of transcranial direct-current stimulation (tDCS) [29, 30], since the electric current tends to flow in directions more parallel to the white matter fiber tracts.

In magnetic stimulation, electric currents inside the tissue are generated by electromagnetic induction, which can also be significantly dispersed by the inhomogeneity and anisotropy of the tissue. During TMS, the electric field and current density distribution induced in the brain were significantly altered by the tissue [27, 31, 32]. Specifically, it was found that tissue types and fiber orientation affect the induced electric field [32] and the outcome of the treatment [27].

#### Impact of cells and cell density on electrical stimulation

The cell membrane is largely non-conductive. Consequently, the presence of the cells will inevitability perturb the externally-applied electric field.

At the single cell level, a spherical cell disturbs the electric potential generated by a microelectrode [33], or a magnetic field [13]. This altered distribution of the microscopic electric field around a single cell could cause secondary effects to the neighboring cells. For example, it will change the membrane potentials in the neighboring cell without direct physical contact between the two cells [34]. Normally, a single cell is surrounded by many other cells. Therefore, although it is possible to predict the polarization and excitability of a single neuron under a specific stimulation electrode arrangement [33], it is nearly impossible to describe the polarization patterns of a single neuron embedded in a large cluster of cells. Indeed, polarization in individual cells depends on the overall density and spatial arrangement of the cells in the three-dimensional cell cluster [35].

#### Impact of impedance change on electrical stimulation at the electrode-tissue interface

When electric currents are delivered by the implanted electrode for neural stimulation, the impedance between the electrode and the contacting tissue determines the electric current flow inside the tissue [36]. However, impedance changes during chronic implantation. The devastated path left by an intracortical-inserted electrode can lead to vascular damage and tearing of the extracellular matrix, damaging both neurons and glial cells [37, 38]. Consequently, increased electrode-tissue impedance is observed in the weeks following electrode implantation in the brain, stabilizing at approximately 3 to 6 months. Postmortem studies from patients confirmed the presence of a fibrous sheath, astrocytosis, neuronal loss, and neuroinflammation in the immediate vicinity of the electrode [39]. Effects of glial scar formation around the electrode also prevent the readout of nerve activity [40]. In a study that investigated the relationship between impedance change and neural recording in a set of chronically implanted animals, the authors found a positive correlation between glial cells and tissue impedance, and a negative correlation between glial cell density and neural signal quality [41]. Establishing how electrical stimulation influences the electrical and histological properties of the surrounding tissue is essential for the development of future neuromodulation systems for more efficient stimulation.

### Cell properties impact neural stimulation with electromagnetic fields

Different cells respond differently to the same electrical stimulation, due to the variabilities in the morphology, biophysical properties, and physiology among different neuronal populations.

#### Variability among neuron types

The nervous system is composed of various types of glia cells and neurons that have separate identities compared to other cells in the system. The outcome of electrical stimulation is dependent on these different types of neurons. For example, the excitability of somatostatin-positive interneurons is higher than that of regular spiking pyramidal neurons in response to various activating stimuli, including extracellular current, low-Mg^2+^/Ca^2+^ artificial cerebrospinal fluid, metabotropic glutamate receptor agonists, and cholinergic agonists [42]. In TMS practice, it was found that there exists select groups of neurons with higher sensitivity to the magnetic field [43]. Indeed, the same magnetic stimulation caused different levels of excitation in layer 5 (L5) pyramidal neurons and interneurons in cortical slices of rats [44].

#### Cell orientation to the electric field

Orientation of the cell to the electric current determines the pattern and intensity of the field-induced membrane potential at the single cell level. This concept was established mostly due to the biophysics modeling of the neuron as a simple geometric shape, such as a spherical cell or spheroidal cell [33, 45, 46]. Numerical modeling with a multi-compartmental method revealed a detailed activation pattern of the targeted neurons with variable orientation. For example, it was reported that clinical DBS electrodes have different effects in axon activation, depending on the axonal orientation. The activation threshold of the axons that were parallel to the electrode was the lowest. As the axons increased their angle toward the perpendicular placement, the activation threshold increased [47].

The biophysics of such orientation-dependent axonal activation has been extensively investigated. Location of activation is largely determined by the gradients of the electric field along the neural tissue, or the activating function [48–50]. Orientations that favor the establishment of large field gradients are essential for electrical activation of the neuron. This theoretical understanding of the impact of orientation on neural activation led to the delicate control of retina ganglion cells by the electric field [51].

Under magnetic stimulation, orientation of the magnetic coil is also a major concern in effective TMS of the motor cortex [7, 11, 12]. At the cellular level, a greater magnetic stimulation is needed when a cell is placed perpendicularly to the magnetically-induced electric field [52, 53]. In an in vitro study, when axons from multiple neurons were forced to form a ring in the culture, the neurons could be magnetically stimulated. In contrast, two-dimensional cultures of comparable size did not respond to magnetic excitation [43]. This happens because the one-dimensional pattern allowed axons in the ring structure to follow the magnetically induced electric field to generate a large field gradient [43].

#### Neural morphology and electrical properties

A typical neuron consists of three major components: the soma, axon, and dendrite. Under electrical stimulation, the basic morphologies of the neuronal elements, including the size and shape of the neural structure, all play a significant role in somatic depolarization by altering the current sources or sinks created by the applied field [54, 55]. Consequently, the geometric and electrical properties of a single neuron affect electrical stimulation.

Computational work revealed that larger cell bodies are associated with greater transmembrane potentials under electrical [56] and magnetic [13] stimulation. The stimulation effects within different brain regions were found to be dependent on the axon fiber size in DBS [57]. A larger axon diameter is associated with a smaller axonal resistance, leading to a lower threshold for excitation.

Similarly, electroporation studies have demonstrated that the threshold for membrane permeabilization is associated with the size of the target cells [58, 59]. The larger cell requires lower external fields to create permeable cell membranes [60]. Computational work also confirms the importance of cell shape to electrical stimulation. Distribution of the induced transmembrane potential under a homogeneous electric field is affected by the shape of the cell. This distribution is more complicated in a spheroidal cell than in a spherical cell [45, 61].

In a typical neuron, the soma leads to the axon through the axon hillock. Placement of electrical stimulation to the neuron, specifically at the initial axonal segment, has resulted in a lower threshold in mouse and rat cortical brain slices. This is due to the great number of voltage gated sodium channels in this area [44, 62]. Axons are further divided regarding the presence or absence of myelin. Myelin sheaths act as insulators, where the thickness, length, and amount of axon coverage greatly affects the stimulation needed. Factors such as the degree of axonal myelination and the presence of large bending axons are known to have an important influence on electrical stimulation [2]. For example, dorsal root ganglion stimulation (DRGS) with electric current was developed in the mid-2010s to treat chronic pain [63]. The stimulation drives the activity of large, myelinated Aβ-fibers, but not small, unmyelinated C-fibers [64]. The last prominent property of the neuron is the dendrite. Dendritic complexity influences electrophysiological characteristics through unusual firing frequency and patterns [65]. The threshold for electrical activation is lower in neurons with symmetrical dendrites than in those with asymmetrical dendrites, due to less axial resistance in the symmetrical dendrites [54].

In addition to the geometric properties of the cell, electrical properties of the cellular environment affect outcomes of electrical stimulation. These biophysical properties include the conductance of the medium, the membrane, and the cytoplasm [66]. Transmembrane potential is dependent on the electric conductance of the media and cytoplasm, and the membrane conductivity under electrical [46] and magnetic stimulation [13, 53, 67]. Medium conductivity also plays a significant role in causing membrane permeability under electroporation [45, 68, 69].

#### Physiological properties of the neurons

Physiological properties of the neurons play compounded roles in electrical stimulation. These include, but are not limited to, the threshold of activation, refractory period, and capability of rebounding after hyperpolarization.

The *threshold* of neural activation is defined as the minimal current needed to excite a neuron [70]. Neurons are more excitable when their membrane potential is just below threshold but not discharging [2]. Similarly, neurons with a low current threshold are more susceptible to magnetic stimulation [44, 52]. The *refractory period* of the neuron puts constraints on the frequency of electrical stimuli for neural activation. There is a limit to the highest frequency of stimulation at which neurons can still respond to individual stimulation pulses.

*Post inhibitory rebound* (PIR) is a phenomenon where a cell will spontaneously fire after it has been intensively inhibited [71, 72]. For example, some inhibitory interneurons in goldfish exhibited a remarkable excitation triggered by a brief hyperpolarization [73]. In clinical practice, this cellular property converts the outcome of electrical stimulation from inhibition to excitation. For example, transcranial alternating current stimulation (tACS) causes a rebound of neural activity, rather than entrenchment [74]. In TMS of the cat primary visual cortex, strong stimuli could lead to an early suppression of activity during the first 100-200 ms, followed by stronger (rebound) facilitation [75]. In DBS, the globus pallidus internus (GPi) and thalamic neuronal activity were initially inhibited, but immediately increased after the stimulation [76]. In DBS on human thalamic neurons, a transient rebound of bursting activity was found, followed by prolonged inhibition after the cessation of the high frequency extracellular stimulation [77]. PIR observed in DBS raised questions about the role of inhibition in the current concepts of basal ganglia physiology [76, 77].

### Dynamic changes in the neurons impact the outcome of electrical stimulation

Neurons are dynamic entities. They grow, mature, and age. Neurons also die in pathological conditions. Neuronal properties could also be altered by electrical stimulation. Activation state of the neuron consistently changes over time. All these dynamic changes will impact the outcome of electrical stimulation.

#### Changes in the biophysical properties and functions of the neurons during development

Neuronal growth can lead to profound effects on the biophysical properties, excitation levels, and function changes of the cells. For example, the electrotonic structure and synaptic integration of the lateral giant neurons in crayfish grow in size during development, leading to decreased input resistance and increased cell membrane constants [78]. These structural and biophysical changes can impact the electrophysiological profile of the brain tissue. For example, certain electric potentials can be generated from the fusiform gyrus in adult brains, but not in infants [79–81]. Neuron excitation also changes during aging. Intracranial electroencephalography (EEG) and spontaneous field potential recordings demonstrate that the overall network activity in the hippocampus increases during aging [82].

Neurons change their functional roles during development. For example, by releasing the neurotransmitter gamma-aminobutyric acid (GABA), GABAergic interneurons are traditionally regarded as inhibitory to network activity. However, GABAergic interneurons can excite and inhibit postsynaptic neurons, depending on the GABA reversal potential in the postsynaptic cells [83, 84]. During early mammalian embryonic development, the level of sodium–potassium-chloride cotransporter 1 (NKCC1) is high, and the level of potassium chloride cotransporter 2 (KCC2) expression is low [85]. This causes a high concentration of intracellular Cl^−^ and depolarization of the reversal potential for GABA (*E*_GABA_). As a result, GABA transmission to these postsynaptic neurons is depolarizing but not hyperpolarizing [86–92].

#### Dynamic changes of the neurons during pathological conditions

Pathological conditions lead to profound changes at the cellular level. In animal models of epilepsy, abnormalities were found in the inhibitory GABAergic neurons [93, 94]. Furthermore, in human temporal lobe epilepsy, the loss of hippocampal interneurons was observed (TLE) [95]. Many neurological diseases cause systemic changes in the axons, such as reduction in internodal lengths [96], segmental demyelination [97], and changes in innervation [98].

Pathophysiological changes in the neurons could alter their excitation states. For example, hemisection of the spinal cord in rats led to changes in synaptic transmission. It caused a reduction in the amplitude and frequency of spontaneous inhibitory postsynaptic currents (sIPSC) in substantia gelatinosa neurons. This led to a change in the overall hyperexcitation state in these neurons [99]. In another example, dendritic sprouting occurred during chronic levodopa (L-DOPA) treatments in parkinsonian rats, leading to a reduction of intrinsic excitability [100].

Ionic gradients can also be altered in pathological conditions. For example, potassium ions are responsible for neuronal excitation and network excitability. During seizure, the ion concentration fluctuated in the neuronal tissue [101].

#### Neuron’s properties change during electrical stimulation

Electric pulses applied to the neuron can change its physiological properties. It is well known that high frequency stimulation protocols lead to long-term potentiation in the synaptic strength [102]. When stimulated with high frequency pulses, axons in the spinal cord demonstrate some “fatigue” responses, manifested as a reduced size of compound action potentials (CAPs) [103]. High frequency electrical stimulation can also change the refractory period of a neuron. Typically, the refractory period lasts about 2 ms, but with high frequency stimulation, the refractory period is extended to 5–10 ms in rat brains. This can be explained by changes in ionic concentrations and channel dynamics [104].

The microscopic ionic environment surrounding a cell changes during electrical stimulation. For example, an increase in potassium concentration was observed in hemiparkinsonian rats that were undergoing DBS [105]. Similarly, excessive potassium ion release happens in the brain slices during electrical stimulation, which leads to the depolarization block of epileptic neurons [106, 107]. Calcium ion concentrations also change during electrical stimulation. Monai et al. [108] demonstrated that tDCS caused synchronous, large-amplitude Ca^2+^ surges across the cortex of transgenic mice.

#### Changes in the activation state of the neurons impacts electrical stimulation

The brain has dynamic internal states [109, 110], which may play a significant role in the outcome of electrical and magnetic stimulation (termed “state-dependent”). For example, electrical stimulation of the ventral tegmental area in rhesus monkeys produced different responses, depending on if the animal was awake or under anesthesia [111]. Similarly, TMS produces different perceptual or behavioral outcomes that may depend on the excitability levels of specific neuronal populations [112]. Recording of extracellular spikes and local field potentials from the cat visual cortex has demonstrated that the outcome of stimulation depends on the pre-TMS state of network activity [113]. The neurobiological mechanisms underlying state-dependent electrical stimulation are largely unknown. Some works have highlighted the importance of neural connectivity underlying state-dependent neural stimulation. Considerable changes have been observed in functional connectivity and correlated activity between the awake state and anesthesia both in monkeys [114] and rodents [115], and animals under these different states have distinct responses to neural stimulation [111].

These observations suggest that stimulation effects could be dependent on the active state of individual neurons. Li et al. found that the neuronal response to electromagnetic stimulation highly depends on the intrinsic cellular dynamics in each state [116]. Yang et al. found that neurons at three different levels of excitation (spiking, bursting, and bistable) demonstrate different dynamic responses under magnetic stimulation. Magnetic stimulation has a minimized impact on the temporal firing sequences of bursting neurons in comparison to the spiking and bi-stable neurons [117]. Clinically, the state of cortical neurons can affect the neuronal response to TMS [118, 119]. Using neurons from the model system *Aplysia californica*, we have found state-dependent neural inhibition by the magnetic field, with neurons firing at a low frequency being more susceptible to inhibition than those firing at a higher frequency [120]. However, as of today, there is a lack of systematic understanding in how stimulation interacts with endogenous neural activity.

### Ephaptic effects between neurons affect electrical stimulation

Neurons use field potentials to communicate, namely by ephaptic effects. By altering the field potential, the exogenously applied electric field could modify neuron behavior by interfering with the ephaptic effects. On the other hand, the ephaptic effects between neurons reshape the efficacy of electrical stimulation.

Ephaptic effects refer to the electrical interactions mediated across the extracellular space. Studies of the goldfish Mauthner cell provided the first clear evidence for such interactions [121]. A single impulse in the M-cell leads to the nearly simultaneous firing of 40 to 80 interneurons nearby [73]. The local electric fields generated by the cooperative action of brain cells can influence the timing of neural activity in the brain [122], and facilitate synchronized, even epileptic-like, neuronal bursting [123] and neuron-glia communication [124]. It is proposed that bioelectric and biomagnetic fields of the astroglial network could equalize extracellular local field potentials (LFPs) and associated local magnetic field potentials (LMFPs) in the cortical layers of the brain. This could contribute to the adequate and coherent integration of external and internal signals in the processing of information [125].

Since ephaptic coupling and electrical stimulation share the same biophysical nature, it is appealing to speculate that the externally applied electric field could interact with the ephaptic coupling between neurons. For example, ephaptic interactions between neurons are known to be a key neuronal mechanism underlying seizure. External electrical stimulation via the interruption of the ephaptic effect is used to suppress seizure [126]. In another example, it was found that nonlinear neuronal oscillators could be coupled indirectly via electromagnetic induction with magnetic flux, through which neurons can communicate without physical connections [127].

On the other hand, ephaptic coupling between neurons could alter the outcome of electrical stimulation. In a computational model of a peripheral nerve trunk, the interstitial space between the fibers and the tissues was modeled using a resistor network, enabling distance-dependent ephaptic coupling between myelinated axons, and between fascicles. Under electrical stimulation, this ephaptic coupling can increase the number of fibers that are activated, reducing the artificial currents required for axonal recruitment. Ephaptic coupling also reduces and shifts the range of optimal stimulation amplitudes for maximum inter-fascicular selectivity [128].

### Molecular changes that affect electrical stimulation

Ion channels are the fundamental units that support neural excitation. Properties of the ion channels shape the output of electrical stimulation. Ion channels change during development, under pathological conditions, and during electrical stimulation. Therefore, the outcome of electrical stimulation may be interpreted in the context of dynamic channel expression and functions.

#### Distribution and properties of ion channels impact electrical stimulation

The mechanisms of neuronal activation by electrical stimulation are largely governed by voltage-gated ion channels. An electrical stimulus leads to membrane potential depolarization and ion flow across the membrane via voltage-gated channels.

Location of voltage sensitive ion channels affects the stimulation. Normally, dendrites (which have lower sodium channel density) have higher activation thresholds than axons of the same diameter in electrical stimulation [129]. A portion of retinal ganglion cell axons, about 40 μm from the soma, demonstrates a specific biophysical property of highly dense sodium channels. Electrode locations close to this band of highly dense sodium channels show the lowest thresholds during electrical stimulation [130].

Variability in different ion channel kinetics affects electrical stimulation. The expression and kinetics of several different voltage-dependent potassium (K_V_) channel subtypes in retina cells play determinant roles in the sensitivity of neurons to electrical stimulation. Consequently, when retina cells are electrically stimulated, the non-spiking amacrine neurons of the retina exhibit a large variety of responses [131].

State of the ion channel affects electrical stimulation. Blockage of voltage-gated potassium (Kv) channels may either increase or decrease cellular excitability, depending on the state of the ion channels. Blocking Kv when the channel is at a closed-state increased the excitability, while a selective block of the open Kv channels decreased the rate of repetitive and consequent Ca influx [132].

#### Ion channels change during development, in pathological conditions, and during electrical stimulation

Mutation of ion channels causes dysfunction in neurons. For example, the action potential initiation mechanism was impaired in GABAergic interneurons in a mouse model that expresses a mutated human Na (V)_1.1_ gene, resulting in a hyperexcitable network [133]. When the functions of voltage-dependent sodium channels were impaired in the GABAergic interneurons, it led to a reduced threshold and accelerated propagation in febrile seizures, and reduced threshold in flurothyl-induced seizures [134].

Ion channels change during neural injury. For example, after spinal cord injury (SCI), dispersed distributions of potassium channel subunit Kv1.1 and Kv1.2 along the injured axons were observed, in contrast to the tight, clustered locations of these channels to the juxtaparanodes of non-injured axons [135].

Electrical stimulation can regulate the expression and dynamics of the ion channels involved in neural excitability. For example, retinal ganglion cells (RGCs) can fire spikes at frequencies greater than 200 Hz when driven by light. RGC spiking rates decreased when electrically stimulated at 50–200 Hz. This depression was caused by a stimulus-frequency-dependent decline of RGC voltage-gated sodium current, mediated by the sodium channels [136].

Electrical stimulation can also regulate the expression and dynamics of the ion channels involved in synaptic transmission. Long-term potentiation (LTP) refers to persistent strengthening of neuronal synapses. It is normally induced by a strong tetanic (high frequency) stimulation. Long-term depression (LTD) refers to the persistent decrease in synaptic strength following weak, low frequency stimulation. AMPA (α-amino-3-hydroxy-5-methyl-4-isoxazolepropionic acid) and NMDA (*N*-methyl-d-aspartate) receptors are permeable to Na^+^ and K^+^ (and Ca^2+^ in NMDARs). The activity of those channels, and the consequent strength of the synapse, depends greatly on the strength and length of stimulation of both presynaptic and postsynaptic neurons [137]. In LTP, more AMPA receptors are inserted into the postsynaptic membrane to potentiate the synapse; in LTD, AMPA receptors are absorbed and removed from the postsynaptic membrane, which weakens the synapse [137]. These neuronal changes, at the ion channel level, result in enhanced or attenuated synaptic transmission triggered by electrical stimulation.

### Lessons learned—neurons have dynamic identities during neuromodulation with electromagnetic stimulations

Converging pieces of evidence have suggested a broader frame of reference in which the interactions between the cell and the electric field define the outcome of electrical stimulation [23]. Specifically, this paper reviews the impact of the dynamic aspects of neurons on the efficacy of stimulation. There are several lessons learned from this study.

#### Importance of improving the biocompatibility of the implants for consistent electrical stimulation

To eliminate inconsistency in neural recording and stimulation due to foreign body reactions, it is essential to improve the biocompatibility of the implants. Next-generation intracortical microelectrodes are being developed with an increased emphasis on reducing the neuro-inflammatory response. The field is actively progressing from traditional, inorganic materials towards approaches that either minimize the microelectrode footprint, or incorporate compliant materials, bioactive molecules, conducting polymers, or nanomaterials with the implants [40]. Biocompatibility can also be improved with drugs. For example, administration of a cell cycle inhibitor (flavopiridol) at the time of surgery would reduce reactive gliosis surrounding neural prostheses. Purcell et al. reported that flavopiridol reduced the expression of a cell cycle protein (cyclin D1) in microglia surrounding the probes, decreased the impedance at 1 kHz, and increased the neural signal [41].

The recent development of micromagnetic stimulation (µMS) technology significantly improves the biocompatibility of the coil implantation. These coils can be manufactured to match the size of the targeted neurons for focal stimulation. They can also be encapsulated under the cover of biocompatible, soft material to avoid the inflammatory and immune responses that occur due to direct contact with the tissue [138, 139]. Evidence has shown that the miniature coil could be used to control retinal ganglion neuron activity with high amplitude pulses [140]. Recently, we reported that µMS could block axonal conductance in unmyelinated axons [141] and in ganglion cells [142]. This technology could also block hyperactive circuit activity in the hippocampus [143].

#### Importance of understanding neural anatomy for efficient and selective stimulation

Since neural morphology plays a significant role in the neuron’s response to electrical stimulation, an in depth understanding of neural anatomy at the microscopic level is essential in the design of efficient protocols for selective stimulation. The importance of neural anatomy includes elements of orientational and directional selectivity. Studies have suggested that these two factors are related in natural firing rates under electrical stimulation [129]. For example, the µMS technology has been used to activate vertically oriented pyramidal neurons (PNs) without activating horizontally oriented passing axons [144]. Achieving such improved selectivity depends on the understanding of brain anatomy, and the theory that the field parallel to the axon is more effective in neural activation than the field that is perpendicular to the axon [145].

#### Importance of monitoring neural activity during electrical stimulation

To ensure electrical stimulation takes effect in the most ideal way, it is essential to use imaging or electrophysiology tools to monitor neural activity changes during the process. This practice is necessary in the execution, evaluation and optimization of electrical stimulation. There are several advantages for such an approach:

- *Ensure precise location for the stimulation electrode* The same stimulation electrode could be used for neural recording from the implanted area, for it is possible to use the shape of neural activity to deduce the location of the extracellular electrodes [146]. This is a reliable strategy to guide the precise positioning of the extracellular electrode, especially when it is challenging to directly monitor the electrode with imaging technology [147]. Rapid predictability based on the waveform shape of the neural activity provides researchers and clinicians with valuable information right from the implantation of an electrode. For example, characteristics of evoked compound action potentials (ECAPs) and local field potentials (LFPs) recorded during DBS provide valuable information between the contact of the electrode and the tissue [148].
- *Reveal any pathological changes in the targeted area* Monitoring neural activity also provides insights to the potential pathological changes in the targeted neural population. Any discrepancies or unexpected shape changes in the neural recording can, therefore, be used to reveal such damage, as seen with strokes [149] and post-concussive syndrome [150]. In another example, acute edema caused a reduction in the single neuron and population ECAP signal energy, as well as LFP magnitude. This information can be used to optimize electrode parameters in a closed-loop DBS system for the treatment of movement disorders [151, 152].
- *Provide predictions of the stimulation outcomes* Since pre-stimulation neural activity is highly related to the post-stimulation outcome [113], it is important to monitor the neural activity prior to stimulation. This technique can generate valuable predictions and a comprehensive understanding of the stimulation outcome.
- *Guide the adjustment of the stimulation protocol* Changes in neural activity can empirically guide the effective use of TMS in both clinical and experimental settings [153]. For example, the instantaneous brain state can be used to promote efficacious plasticity induction by TMS [154]. Combining TMS with functional magnetic resonance imaging (fMRI) is powerful in revealing if different TMS intensities are needed to induce neural activation [155].

#### Importance of utilizing the dynamic state of the neurons for efficient neural stimulation

Because the dynamic changes at the cellular level impact the outcome of electrical stimulation, it is essential to apply state-dependent brain stimulation to match and compensate for the state changes.

- *Preconditioning the brain state for efficient stimulation* It is essential to develop technology that can precondition the state of the neural network to enhance the stimulation outcome [156]. For example, tDCS was used to precondition the motor cortex for a better outcome in low-frequency rTMS [157, 158]. In another example, transcranial random noise stimulation (tRNS) was used to precondition the primary motor cortex. This led to a lower response threshold and increased responsiveness when probed with TMS [159].
- *Adjusting the brain state with pharmacology for efficient stimulation* It is possible to consider using a pharmacological approach to alter the excitability of the nervous system and maximize the clinical outcome of rTMS. As a proof, when anticonvulsant phenytoin was administrated, the magnetic field was more effective in decreasing audiogenic seizure severity in mice [160, 161]. Similarly, bursts of high frequency rTMS, together with lorazepam, suppressed seizures in a rat kainate status epilepticus model [162], with the combined methods more effective than rTMS alone. Future research should explore the possibility of improving complementary therapies by adjusting the excitability state of the nervous system with drugs.

#### Importance of developing closed-loop stimuli to adapt to the quick changes in neural activity

To apply fast, purpose-driven stimulation to compensate for the dynamic changes in the brain, new technology should explore real-time, closed-loop measurement and stimulation paradigms. Real-time, multi-channel EEG data can be used to monitor the brain state online and modify stimulation parameters [163] to apply state-dependent brain stimulation. EEG can also be used to design closed-loop, purpose-driven stimuli to provide brain-state guided stimulation [164, 165]. For example, Ganzer et al. [166] used closed-loop vagus nerve stimulation (CLVS) to enhance recovery after SCI. To generate the best control, the authors developed a real-time, closed-loop neuromodulation paradigm based on the synaptic eligibility trace, to deliver CLVS immediately after the most successful forelimb movements during motor rehabilitation.

#### Importance of including neural dynamics in computer simulations of neuromodulation

To appreciate the complexity and reveal the underlying mechanisms of electrical stimulation, researchers use computer simulation to address large sets of parameters and variabilities. The typical stimulation protocol includes a three-step simulation approach [70, 167–169]. First, the electric current distribution generated by an electrode/coil is computed in the 3D space. Second, a multi-compartment neural model is built to represent the fine, geometric structure of the neuron or neural networks, with channel mechanisms incorporated into each component. Finally, the electric field obtained from the first step is used to activate the neurons. This classic framework of computation assumes, first, that the extracellular electric field is always treated equally around each model compartment. Second, the extracellular electric field is not affected by the presence of the tissue. Third, the extracellular voltage generated by the membrane current is neglected. Under these assumptions, the extracellular electric field is computed without considering the existence of the tissue, or its counter effect to the externally-applied electric fields. Although such an approach is typical in the field [23, 167], ignorance of the presence of the neuron and its dynamics could potentially cause underestimation of the applied electric fields, and introduce potential inaccuracies in the modeling [33, 170].

Evidence provided in this review paper further suggests that all three aspects of this modeling approach should be improved. Since tissue inhomogeneities play significant roles in the distribution of the externally applied electrical stimulation, a precise understanding of the electric field distribution in the context of the complicated, dynamic tissue properties is essential in studying neural stimulation with the electric field. Computational tools, such as finite element modeling (FEM), which can delineate the tissue complexity and represent the resistive and capacitive properties of the tissue at the micro scale [171], will be appealing. A successful example is the model study on parkinsonian symptoms with electrical stimulation of the subthalamic nucleus (STN). Sotiropoulos and Steinmetz [57] included tissue inhomogeneity and anisotropy in the simulation. The model successfully validated that the tissue’s properties (i.e., degree of inhomogeneity and anisotropy) have a direct impact on stimulation effects, and that increasing STN conductivity could cause neuronal activation outside the STN.

We must consider the neuron as a dynamic entity that consistently changes in anatomy and variable excitation states. Some recent developments of computational technology have begun to address these shortcomings. As the first step, the impact of the presence of the neuron must be better represented in the model. For example, a “whole finite element” approach allows the simultaneous calculation of the extracellular and intracellular potentials by representing the neuronal membrane with a thin-film approximation [167]. This approach illustrates the difference of the neuronal response between the sides of the soma membrane either facing or opposite to the stimulating electrode. By simultaneous calculation of the stimulation potential and the neuron response, this novel approach provides a mechanism to better represent the neuron and its dynamic behavior during electrical stimulation. These improvements result in more accurate results that more closely match the biological reality.

## Conclusions

Neurons are dynamic elements in the practice of neuromodulation with electromagnetic stimulation. Ultimately, the outcome of neural stimulation depends on the complicated interactions between the dynamic neurons and the externally applied electric field. Therefore, the neuron matters when we consider the outcomes of electrical stimulation in both laboratory research and in clinical practice. Further research should consider the dynamic properties of neurons in interpreting experimental data and incorporate cell-field interactions when setting up computer simulation.

## Data Availability

Data sharing is not applicable to this article as no datasets were generated or analyzed during the current study.

## Abbreviations

AMPA: α-Amino-3-hydroxy-5-methyl-4-isoxazolepropionic acid
CAPs: Compound action potentials
CLVS: Closed-loop vagus nerve stimulation
DBS: Deep brain stimulation
DRGS: Dorsal root ganglion stimulation
ECAPs: Evoked compound action potentials
EEG: Electroencephalography
*E*_GABA_: Reversal potential of GABA
FEM: Finite element modeling
fMRI: Functional magnetic resonance imaging
GABA: Gamma-aminobutyric acid
KCC2: Potassium chloride cotransporter 2
L-DOPA: Levodopa
LFPs: Local field potentials
LMFPs: Local magnetic field potentials
LTD: Long-term depression
LTP: Long-term potentiation
µMS: Micromagnetic stimulation
NKCC1: Sodium–potassium-chloride cotransporter 1
NMDA: *N*-Methyl-d-aspartate
PIR: Post inhibitory rebound
PNs: Pyramidal neurons
RGCs: Retinal ganglion cells
rTMS: Repetitive transcranial magnetic stimulation
SCI: Spinal cord injury
SCS: Spinal cord stimulation
sIPSC: Spontaneous inhibitory postsynaptic currents
STN: Subthalamic nucleus
tACS: Transcranial alternating current stimulation
tDCS: Transcranial direct-current stimulation
TLE: Temporal lobe epilepsy
TMS: Transcranial magnetic stimulation
tRNS: Transcranial random noise stimulation
